# Use of SNP chips to detect rare pathogenic variants: retrospective, population based diagnostic evaluation

**DOI:** 10.1136/bmj.n214

**Published:** 2021-02-15

**Authors:** Weedon MN, Jackson L, Harrison JW, Ruth KS, Tyrrell J, Hattersley AT, Wright CF

**Affiliations:** Institute of Biomedical and Clinical Science, University of Exeter College of Medicine and Health, Royal Devon and Exeter Hospital, Exeter EX2 5DW, UK

## Abstract

**Objective:**

To determine whether the sensitivity and specificity of SNP chips are adequate for detecting rare pathogenic variants in a clinically unselected population.

**Design:**

Retrospective, population based diagnostic evaluation.

**Participants:**

49 908 people recruited to the UK Biobank with SNP chip and next generation sequencing data, and an additional 21 people who purchased consumer genetic tests and shared their data online via the Personal Genome Project.

**Main outcome measures:**

Genotyping (that is, identification of the correct DNA base at a specific genomic location) using SNP chips versus sequencing, with results split by frequency of that genotype in the population. Rare pathogenic variants in the *BRCA1* and *BRCA2* genes were selected as an exemplar for detailed analysis of clinically actionable variants in the UK Biobank, and BRCA related cancers (breast, ovarian, prostate, and pancreatic) were assessed in participants through use of cancer registry data.

**Results:**

Overall, genotyping using SNP chips performed well compared with sequencing; sensitivity, specificity, positive predictive value, and negative predictive value were all above 99% for 108 574 common variants directly genotyped on the SNP chips and sequenced in the UK Biobank. However, the likelihood of a true positive result decreased dramatically with decreasing variant frequency; for variants that are very rare in the population, with a frequency below 0.001% in UK Biobank, the positive predictive value was very low and only 16% of 4757 heterozygous genotypes from the SNP chips were confirmed with sequencing data. Results were similar for SNP chip data from the Personal Genome Project, and 20/21 individuals analysed had at least one false positive rare pathogenic variant that had been incorrectly genotyped. For pathogenic variants in the *BRCA1* and *BRCA2* genes, which are individually very rare, the overall performance metrics for the SNP chips versus sequencing in the UK Biobank were: sensitivity 34.6%, specificity 98.3%, positive predictive value 4.2%, and negative predictive value 99.9%. Rates of BRCA related cancers in UK Biobank participants with a positive SNP chip result were similar to those for age matched controls (odds ratio 1.31, 95% confidence interval 0.99 to 1.71) because the vast majority of variants were false positives, whereas sequence positive participants had a significantly increased risk (odds ratio 4.05, 2.72 to 6.03).

**Conclusions:**

SNP chips are extremely unreliable for genotyping very rare pathogenic variants and should not be used to guide health decisions without validation.

## Introduction

Single gene disorders are usually caused by genetic variants that are very rare in the population (<1 in 10 000 people).^1^ Finding one of these rare pathogenic variants confers a high probability of disease in an individual and their family that requires referral for clinical follow-up. For example, a confirmed pathogenic variant in one of the breast cancer genes *BRCA1* or *BRCA2* would need urgent follow-up with additional screening and potentially prophylactic surgical mastectomy and oophorectomy.^2^ Molecular diagnostic laboratories typically use highly accurate DNA sequencing technologies to test for these types of rare pathogenic variants.^3^ ^4^

**Fig 1:**
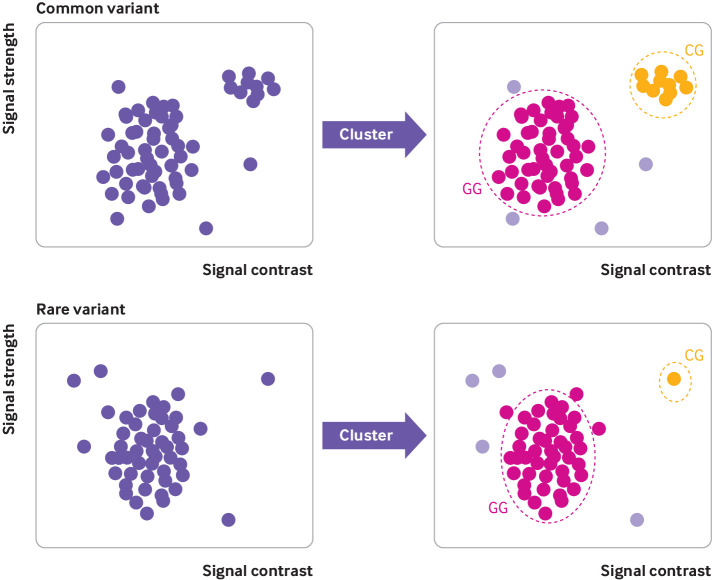
Explanation of genotyping using SNP chip technology. Example cluster plots for common variant (top) and rare variant (bottom). Each circle represents one person’s DNA assayed at specific position on SNP chip when known variant (G to C) exists. Automated clustering across multiple individuals is used to determine which DNA base is present in each person at that position. Pink circles in main cluster represent most common reference base (G), orange circles represent heterozygous variant (C), and pale purple circles represent uncertain or missing results due to experimental noise

SNP chips are DNA microarrays that test genetic variation at many hundreds of thousands of specific locations across the genome.^5^ They were initially designed for testing single nucleotide polymorphisms (SNPs) that are common in the population (>1 in 100 people). SNP chips have proven to be excellent for studying common genetic variation, which can be used to assess ancestry,^6^ as well as predisposition to many complex multifactorial diseases such as type 2 diabetes.^7^ ^8^ The genetics community generally recognises that SNP chips perform poorly for genotyping rare genetic variants owing to their reliance on data clustering (fig 1).^9^ ^10^ ^11^ Clustering data from multiple individuals with similar genotypes works very well when variants are common, as large numbers of data points are available (fig 1, top). However, clustering becomes more difficult as the number of people with a particular genotype decreases; most people will have the reference genotype (normal allele), and distinguishing an alternative genotype (variant allele) from experimental noise is extremely difficult when only a single carrier is present (fig 1, bottom).

**Fig 2:**
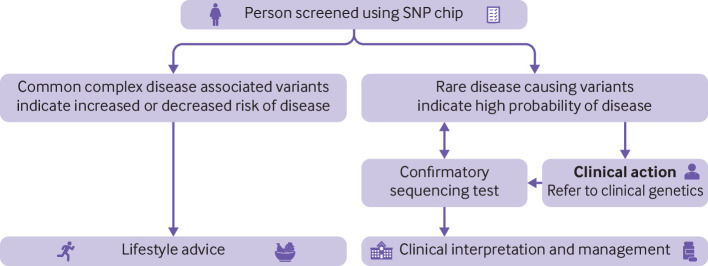
Current medical context of SNP chip screening

Despite this problem, in recent years many SNP chip designs, including those used by many direct to consumer companies, have been augmented to include rare pathogenic variants that cause single gene disorders. As a consequence, consumers of such tests are increasingly being screened for many rare single gene disorders and are potentially receiving medically actionable results, for which they often seek advice from healthcare professionals (fig 2).^12^ False positive results for rare clinically actionable variants detected by direct to consumer SNP chips have been described in practice guidance,^13^ several case reports,^14^ ^15^ and two small case series.^16^ ^17^ However, no systematic evaluation of the performance of SNP chips for assaying rare genetic variants has been published. It has been estimated that more than 26 million people had accessed direct to consumer genetic testing at the start of 2019,^18^ so knowing how accurate these results are is likely to be is crucial in order to interpret rare pathogenic variants detected using SNP chips.

In this study, we used sequencing data from 49 908 UK Biobank participants as a reference standard to do a large scale, systematic evaluation of how well SNP chips detect rare genetic variants.^10^ We sought to answer two questions. (1) How well do SNP chips perform at detecting pathogenic genetic variants in individuals compared with the sequencing data in the UK Biobank? (2) Are the sensitivity and specificity of SNP chips adequate for rare variants? We used rare pathogenic variants in the *BRCA1* and *BRCA2* genes (collectively termed BRCA henceforth) that cause hereditary cancers as an exemplar to evaluate the performance of SNP chips in the UK Biobank for genotyping clinically actionable variants. We replicated our findings using data from 21 people who had had direct to consumer genetic testing and shared their data online via the Personal Genome Project.^19^

## Methods

### Study design, participants, and test methods

We did a retrospective comparison of SNP chip genotyping (index test) with next generation sequencing (here defined as the reference standard) from the UK Biobank and Personal Genome Project participants for whom both datasets were available. We studied 49 960 individuals (55% female) from the UK Biobank. The UK Biobank is a population based research cohort of approximately 500 000 participants recruited in the UK between 2006 and 2010. Approximately 9.2 million people aged 40-69 years who lived within 40 km of one of 22 assessment centres were invited and 5.5% participated.^10^ The Personal Genome Project is a community project in which participants are invited to publicly share their genetic data.^19^ We compared variants genotyped by using SNP chips (index test) with next generation sequencing data (reference standard) in the same individual.

### UK Biobank

Next generation exome sequencing data were available on 49 960 participants, of whom 49 908 also had SNP chip data that had passed quality control. SNP chip data were generated centrally by the UK Biobank, and the exome sequencing data were generated externally by Regeneron and returned to the UK Biobank resource as part of an external access application request.^20^ A subset of 4037 participants were previously genotyped using the Applied Biosystems UK BiLEVE Axiom Array by Affymetrix (807 411 genetic markers), and the other 45 871 participants were previously genotyped using the Applied Biosystems UK Biobank Axiom Array (825 927 genetic markers) that shares 95% of its marker content with the BiLEVE.^10^ Participants were genotyped in 106 batches of around 5000 samples. We included samples that passed central UK Biobank quality control on either of the UK Biobank SNP chips and used standard quality metrics to exclude problematic SNPs (missingness rate <5% and Hardy Weinberg P<1×10^−6^).^11^ We used the UCSC genome browser liftover tool to convert SNP chip variant positions that were reported in human genome build 37 to 38 coordinates for direct comparison with sequencing data.

### Personal Genome Project

We analysed publicly available datasets within the Personal Genomes Project (https://my.pgp-hms.org/) to determine which individuals had both direct to consumer SNP chip and sequencing data in genome build 37. We subsequently downloaded SNP chip data (provided by 23andMe from 2012 to 2019 using Illumina arrays) and genome sequencing data (provided by Veritas Genetics) for 21 people.

### Analyses

We compared variants directly genotyped on the SNP chips with the equivalent positions in the sequencing data from the same individual, excluding sites that were not well sequenced. For genome-wide comparison with SNP chip genotypes, we included only directly genotyped single nucleotide variants with genomic positions present in the genomic Variant Call Format (gVCF) files and covered by more than 15 reads in the sequencing data. For UK Biobank data, we used the minor allele frequency from all 488 377 SNP chip genotyped UK Biobank participants.^10^ For Personal Genome Project data, we used VCF converter to analyse whether variants detected by the SNP chip were present in the sequence data, and we used the minor allele frequencies from gnomAD and the 1000 genomes project.^21^ ^22^ We tested the genotyping quality of each individual variant on the SNP chips versus sequencing and calculated average performance metrics per variant overall and for common and rare variant subsets in the UK Biobank.

For detailed gene specific comparison with SNP chip genotypes in the UK Biobank, we included directly genotyped single nucleotide variants, as well as small insertions and deletions in the *BRCA1* and *BRCA2* genes. We defined variants as pathogenic if they were predicted to result in a truncated protein or had previously been classified as likely or definitely pathogenic in the ClinVar database^23^; we included variants with conflicting reports of pathogenicity that included pathogenic assertions made within the previous five years. We visually examined sequencing data with the Integrative Genomics Viewer for all index and reference positive results to determine whether the variant was present.^24^ We extracted cancer registry data for breast, ovarian, prostate, and pancreatic cancer for all participants in April 2019 to coincide with the most recent release of Hospital Episode Statistics data. We used logistic regressions to assess the relation between participants who tested positive and any BRCA related cancer. We included age at cancer registry, sex, recruitment centre, death, breast screening, and non-cancer related mastectomy in the regression.

Results are presented in accordance with the standard framework for the validation and verification of clinical molecular genetic tests and STARD guidelines for reporting diagnostic accuracy studies, using sensitivity, specificity, positive predictive value, and negative predictive value to evaluate assay performance.^25^ ^26^

### Patient and public involvement

Patients and the public were not directly involved in the design or implementation of this study, as we used previously generated data. As part of the consent process for the UK Biobank, National Health Service patients gave their consent for the collection, storage, and use of their genetic data and medical records by all approved researchers. All of the participant records are linked-anonymised.

## Results

### Performance of SNP chips for all variants

In the 49 908 UK Biobank participants, we compared genotypes for 108 574 single nucleotide variants that were classified as heterozygous by the SNP chip to reference standard sequencing data. Of the 49 908 participants, 45 871 were genotyped using the Axiom chip and 4037 using the BiLEVE chip. Overall performance across both chips for all variants was very good (table 1), with 3.1×10^8^ true positives, 4.6×10^9^ true negatives, 3.2×10^6^ false positives, and 2.7×10^6^ false negatives. Performance for genotyping common SNPs with a frequency of more than 1% was especially good (table 1), both for the Axiom chip (average sensitivity 99.8%, specificity 99.7%, positive predictive value 99.0%, and negative predictive value 99.9%) and the BiLEVE chip (average sensitivity 99.7%, specificity 99.7%, positive predictive value 98.7%, and negative predictive value 99.9%).

**Table 1:** Performance of UK Biobank SNP chips versus sequencing for protein coding variants in UK Biobank

SNP chip and dataset	Sensitivity, % (95% CI)	Specificity, % (95% CI)	Positive predictive value, % (95% CI)	Negative predictive value, % (95% CI)
UK Biobank Axiom (n=45 871):				
All exome variants	96.3 (96.2 to 96.4)	99.9 (99.9 to 99.9)	85.9 (85.7 to 86.1)	99.9 (99.9 to 99.9)
MAF>1%	99.8 (99.8 to 99.8)	99.7 (99.7 to 99.8)	99.0 (98.9 to 99.1)	99.9 (99.9 to 99.9)
MAF<0.001%	29.5 (27.4 to 31.5)	99.9 (99.9 to 99.9)	16.1 (14.9 to 17.3)	99.8 (99.8 to 99.8)
UK Biobank BiLEVE (n=4037):				
All exome variants	96.9 (96.8 to 97.0)	99.9 (99.9 to 99.9)	91.1 (91 to 91.2)	99.9 (99.9 to 99.9)
MAF>1%	99.7 (99.7 to 99.8)	99.7 (99.7 to 99.8)	98.7 (98.6 to 98.8)	99.9 (99.9 to 99.9)
MAF<0.001%	4.4 (3.1 to 5.8)	99.9 (99.9 to 99.9)	9.4 (6.8 to 12.0)	99.7 (99.7 to 99.8)

Results are split by SNP chip and variant group: all exome variants, common variants (MAF >1%) and very rare variants (MAF <0.001%). Results are based on simple mean and standard error of each metric across all relevant single nucleotide variants; insertions and deletions are excluded.

MAF=minor allele frequency.

### Performance of SNP chips in relation to allele frequency

**Fig 3:**
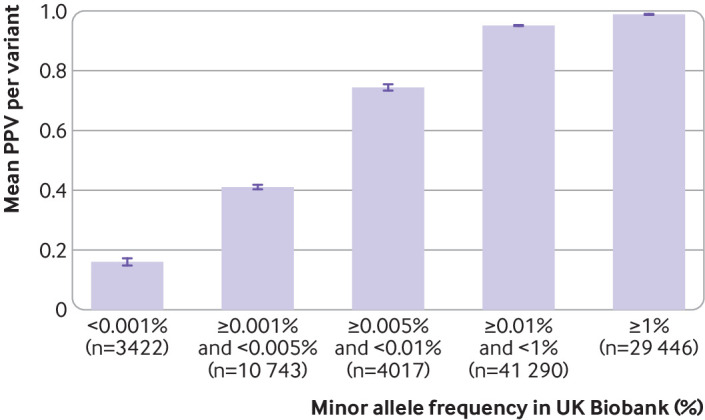
Positive predictive value (PPV) of UK Biobank Axiom SNP chip for detecting variants at different population frequencies. Similar trend was seen with UK Biobank BiLEVE chip (supplementary figure A) and Personal Genome Project consumer data (supplementary figure B)

The genotyping performance of the SNP chips in the UK Biobank was strongly related to the frequency of the variant in the population (table 1; fig 3; supplementary figure A). We found 10 891 (Axiom) and 7408 (BiLEVE) variants on the two UK Biobank SNP chips with a frequency below 0.001% that were also genotyped by exome sequencing in the UK Biobank. For these very rare variants, the sensitivity of the SNP chips to detect heterozygous genotypes was low (29.5% for Axiom and 4.4% for BiLEVE). However, because of the large excess of true negatives, the specificity and negative predictive value both remained high (>99.7% for both chips). The positive predictive value was also strikingly reduced for rare variants compared with common variants—that is, we found a very high proportion of false positives for which the SNP chip (index test) detected a variant allele that was not present in the sequence data (reference standard). For the very rare variants present in UK Biobank, including 4757 heterozygous genotypes across both SNP chips, only 16.1% of Axiom SNP chip heterozygous genotypes (708 true positives in 4239 participants at 3422 variants) were confirmed by the sequencing data, as were only 9.4% (46 true positives in 518 participants at 488 variants) for the BiLEVE chip. We observed a similar performance for very rare variants in the Personal Genome Project data (supplementary figure B), with a positive predictive value of 14% for variants with a population frequency below 0.01% in 21 people (83 true positives at 594 variants).

### Performance of SNP chips for rare pathogenic variants

**Fig 4:**
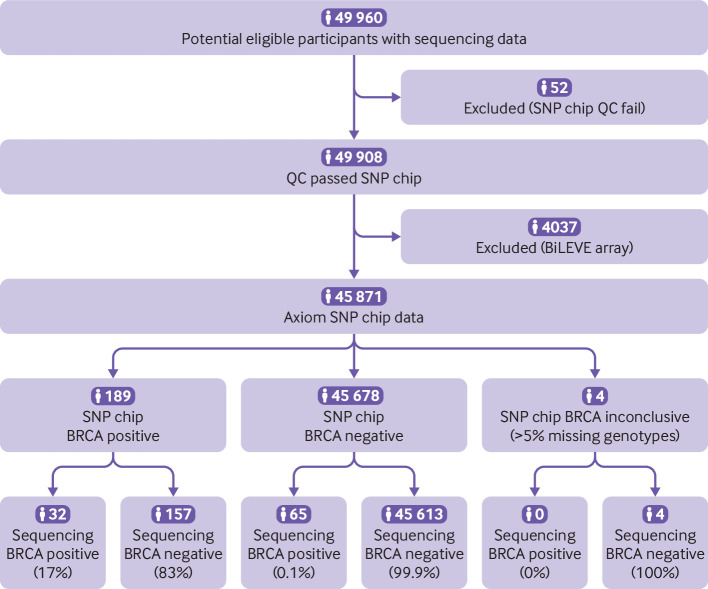
STARD diagram to report flow of participants with pathogenic BRCA variant on UK Biobank Axiom chip compared with sequencing. Similar process was followed for BiLEVE chip (supplementary figure C). QC=quality control

We went on to evaluate the performance of the SNP chips in the UK Biobank for 1139 pathogenic and likely pathogenic variants in BRCA that were included on the chips (fig 4; supplementary figure C); 916 (80%) of these are rare with an allele frequency below 0.01% in the UK Biobank. The performance of both chips was very poor for genotyping pathogenic BRCA variants: overall sensitivity 34.6%, specificity 98.3%, positive predictive value 4.2%, and negative predictive value 99.9% (table 2). Across both SNP chips, 425 pathogenic BRCA variants were detected in 889 UK Biobank participants. Of these, just 17 variants in 37 participants were present in the sequencing data, and the others were false positives; the most common true positive was present in 10 participants and had conflicting and uncertain interpretations in ClinVar. A further 43 pathogenic BRCA variants were present in the sequencing data of 70 participants but were not detected by either SNP chip despite being assayed. The performance of both chips for genotyping pathogenic BRCA variants was very poor (table 2): sensitivity 33.0% and specificity 99.7% for the Axiom chip; sensitivity 50.0% and specificity 82.7% for the BiLEVE chip.

**Table 2:** Performance of UK Biobank SNP chips versus sequencing for all BRCA pathogenic variants in UK Biobank

SNP chip	True positive	False positive	False negative	True negative	Sensitivity, % (95% CI)	Specificity, % (95% CI)	PPV, % (95% CI)	NPV, % (95% CI)
UK Biobank Axiom (n=45 871)	32	157	65	45 613	33.0 (23.8 to 43.3)	99.7 (99.6 to 99.7)	16.9 (12.9 to 22.0)	99.9 (99.9 to 99.9)
UK Biobank BiLEVE (n=4037)	5	695	5	3321	50.0 (18.7 to 81.3)	82.7 (81.5 to 83.9)	0.7 (0.4 to 1.3)	99.9 (99.7 to 99.9)

Results are split by SNP chip; single nucleotide variants, insertions, and deletions are included, and all sequence positive variants were visually confirmed.

NPV=negative predictive value; PPV=positive predictive value.

Risk of BRCA related hereditary cancer in UK Biobank participants with a pathogenic BRCA variant genotyped by SNP chips was similar to the risk of those without an SNP chip BRCA variant. As expected, given the low positive predictive value, the risk of BRCA related cancers in UK Biobank participants with a positive SNP chip result for any pathogenic BRCA variant was similar to the age matched risk in the UK Biobank (odds ratio 1.31, 95% confidence interval 0.99 to 1.71). In contrast, those with a positive sequencing result—including the 107 participants with BRCA variants assayed by either chip, plus another 137 with pathogenic BRCA variants not assayed by either chip—had a markedly increased risk (odds ratio 4.05, 2.72 to 6.03).

We also investigated rare pathogenic and likely pathogenic variants in the Personal Genome Project, where the SNP chips used were made by a different manufacturer and based on a different design than those used in the UK Biobank. Across 21 individuals, 100% (47/47) of rare (minor allele frequency <0.01%) pathogenic single nucleotide variants and 74% (25/34) of likely protein truncating insertions and deletions in known disease causing genes were incorrectly genotyped by the SNP chips. These included a pathogenic variant in *KCNQ2* that causes early infantile epileptic encephalopathy and protein truncating variants in *MSH2* and *MSH6* that confer a very high risk of colorectal cancer.^27^ ^28^ A rare pathogenic variant in *ABCC8* that causes congenital hyperinsulinism was incorrectly genotyped as homozygous in 43% (9/21) of individuals.^29^ Overall, 95% (20/21) of people investigated had a least one false positive rare pathogenic variant compared with sequencing.

## Discussion

We have shown that SNP chips are extremely poor for correctly genotyping very rare variants compared with sequencing data and that, for an individual person, a positive result for a very rare pathogenic variant is more likely to be wrong than right. This finding can be explained as follows. An individual rare variant is very unlikely to be present in any clinically unselected individual, so most results for that variant are true negatives. However, because SNP chips typically assay many thousands of rare variants simultaneously, and have a specificity that is less than 100%, false positive results will occur and outnumber true positives across all rare variants. Any individual person is therefore more likely to have a false positive result across all the rare variants than a true positive result at that variant.

### Strengths and limitations of study

We present the largest evaluation to date of the quality of genotyping for rare variants, using retrospective data from the UK Biobank. Although our analyses were limited by the array designs used in the UK Biobank, our results reflect a fundamental property of SNP chip technology, so we expect our findings to be broadly applicable to most SNP chip datasets. SNP chips are widely recognised as not being good at genotyping very rare variants.^9^ ^10^ ^11^ Some more recent SNP chips have been designed to include only low and intermediate frequency coding variants (>1 in 5000), for which the SNP chips perform relatively well. However, SNP chips are increasingly being augmented with very rare pathogenic variants, which, as we have shown, are not well genotyped. This is an inherent problem of using SNP chip technology to genotype very rare variants and is caused by both the rarity of the variant and the data clustering method on which SNP chips rely (fig 1). Relying on data clustering means that both variant frequency and batch size will affect accuracy, with fewer individuals per batch leading to more genotyping errors for rare variants. As a result, although the performance of chips from different manufacturers may differ owing to different underlying chemistries,^5^ our findings are likely to be generalisable to most SNP chips, and the results are strikingly similar across different SNP chips used in both our UK Biobank and Personal Genome Project datasets. Sequencing is not affected by the same technical problem as SNP chips and thus provides a much more accurate method for genotyping rare variants.^30^

### Policy implications

The problem of SNP chips incorrectly genotyping very rare variants can be partially remedied through improved probe design, removal of poorly performing probes, using custom variant detection definitions,^31^ using multiple probes for individual variants, or adding positive laboratory controls to improve clustering of variants. Many consumer genetic testing companies use these additional quality control methods, supplemented by validation of important variants through DNA sequencing, to improve the accuracy of variants they advertise and report directly to consumers. However, most direct to consumer companies (including those focused on ancestry or other non-medical traits) also allow customers to download and analyse their raw data, which will often include many thousands of additional rare variants assayed on their SNP chip that have not undergone stringent quality control and are therefore much more likely to be false positives.^15^ A recent study found that 89% of consumers of genetic tests downloaded their raw data and 94% of those used at least one third party interpretation service to analyse the results.^32^ We have therefore focused on the direct to consumer scenario because errors in raw SNP chip data could cause significant harm to consumers without further validation. However, erroneous results from SNP chips used in research biobanks can also lead to false associations and wasted resource in the development of new treatments against the wrong targets.^33^ ^34^ ^35^

The inherent technical limitation of SNP chips for correctly detecting rare genetic variants is further exacerbated when the variants themselves are linked to very rare diseases. As with any diagnostic test, the positive predictive value for low prevalence conditions will necessarily be low in most individuals. For pathogenic BRCA variants in the UK Biobank, the SNP chips had an extremely low positive predictive value (1-17%) when compared with sequencing. Were these results to be fed back to individuals, the clinical implications would be profound. Women with a positive BRCA result face a lifetime of additional screening and potentially prophylactic surgery that is unwarranted in the case of a false positive result. Conversely, although the false negative rate of SNP chips is generally low, many very rare pathogenic variants are not included in the design and will therefore be missed.^36^ Women who receive a false negative BRCA result but have a strong family history of breast and/or ovarian cancer are at high risk of developing cancer that could be greatly reduced through preventive surgeries and other interventions.^37^

### Conclusions

Using a large population research cohort and a small consumer genetic testing cohort, we have shown that positive results from SNP chips for very rare variants are more likely to be wrong than right. We therefore urge clinicians to validate any SNP chip results from direct to consumer companies or research biobanks by using a standard diagnostic test before recommending any clinical action. In addition, people with symptoms or a family history of breast and/or ovarian cancer who have received a negative SNP chip result should not be reassured that their risk is low,^13^ and standard referral guidelines should be followed for diagnostic testing (see https://cks.nice.org.uk/breast-cancer-managing-fh). We suggest that, for variants that are very rare in the population being tested, genotyping results from SNP chips should not be routinely reported back to individuals or used in research without validation. Clinicians and researchers should be aware of the poor performance of SNP chips for genotyping very rare genetic variants to avoid counselling patients inappropriately or investing limited resources into investigating false associations with badly genotyped variants.

**What is already known on this topic**

SNP chips are an accurate and affordable method for genotyping common genetic variants across the genome
They are often used by direct to consumer genetic testing companies and research studies
However, several case reports suggest that they perform poorly for genotyping rare genetic variants when compared with sequencing

**What this study adds**

This study, using large scale SNP chip and sequencing data from the UK Biobank, confirms that SNP chips are highly inaccurate for genotyping rare, clinically actionable variants
SNP chips had a very low positive predictive value of less than 16% for detecting very rare variants; most variants with population frequency below 0.001% were false positives
Very rare variants assayed using SNP chips should not be used to guide health decisions without validation

**Glossary**

*Allele*: each of two or more alternative forms of DNA that are found at the same location on a chromosome

*Exome*: ~1-2% of the human genome that codes for proteins

*Genotyping*: method for determining the base (A, G, T, or C) present at a specific location in a person’s DNA

*Heterozygous*: two different alleles in an individual

*Homozygous*: two identical alleles in an individual.

*Negative predictive value*: proportion of normal alleles found by the index test that are confirmed by the reference standard (true negative/(true negative + false negative))

*Positive predictive value*: proportion of variant alleles found by the index test that are confirmed by the reference standard (true positive/(true positive + false positive))

*Sensitivity*: proportion of variant alleles detected by the reference standard that are also found by the index test (true positive/(true positive + false negative))

*Sequencing*: method for determining the order of bases in a DNA sample

*Single gene disorder*: disease caused by, or with a high probability of developing due to, a rare genetic variant in a specific single gene

*Single nucleotide polymorphism* (SNP): type of single nucleotide variant that is common and present in more than 1% of the population (pronounced “snip”)

*SNP chip*: DNA microarray that is used to genotype known genetic variants (typically SNPs) in the population

*Specificity*: proportion of normal alleles detected by the reference standard that are also found to be normal by the index test (true negative/(true negative + false positive))

*Variant* (single nucleotide variant): position in the genome where an individual differs from the reference human genome by a single base change (ie, a substitution of a single letter of DNA). A variant may be rare or common in the population

## Web extra

Extra material supplied by authors

Web appendix: Supplementary figures

## Notes

Contributors: MNW and LJ contributed equally to this work. CFW, MNW, and ATH designed the study. CFW wrote the first draft of the manuscript. All authors reviewed and edited the manuscript. MNW, LJ, JWH, KSR, JT, ATH, and CFW were involved in data processing, statistical analysis, and interpretation. The corresponding author attests that all listed authors meet authorship criteria and that no others meeting the criteria have been omitted. MNW and CFW are the guarantors.

Ethical approval: The UK Biobank has received ethics approval from the National Health Service National Research Ethics Service (ref 11/NW/0382).

The lead author (the manuscript’s guarantor) affirms that this manuscript is an honest, accurate, and transparent account of the study being reported; that no important aspects of the study have been omitted; and that any discrepancies from the study as planned (and, if relevant, registered) have been explained.

Data sharing: The data reported in this paper are available via application directly to the UK Biobank. Direct to consumer data are available from the Personal Genome Project website.

Dissemination to participants and related patient and public communities: We will disseminate this research through the UK Biobank network. The Personal Genome Project participants have freely shared their data online to be used by the research community.

Provenance and peer review: Not commissioned; externally peer reviewed.

## References

[1] Whiffin N Minikel E Walsh R. Using high-resolution variant frequencies to empower clinical genome interpretation. Genet Med 2017;19:1151-8. 10.1038/gim.2017.26 28518168 PMC5563454

[2] AlHilli MM Al-Hilli Z. Perioperative Management of Women Undergoing Risk-reducing Surgery for Hereditary Breast and Ovarian Cancer. J Minim Invasive Gynecol 2019;26:253-65. 10.1016/j.jmig.2018.09.767 30240898

[3] Clark MM Stark Z Farnaes L. Meta-analysis of the diagnostic and clinical utility of genome and exome sequencing and chromosomal microarray in children with suspected genetic diseases. NPJ Genom Med 2018;3:16. 10.1038/s41525-018-0053-8 30002876 PMC6037748

[4] Ellard S Kivuva E Turnpenny P. An exome sequencing strategy to diagnose lethal autosomal recessive disorders. Eur J Hum Genet 2015;23:401-4. 10.1038/ejhg.2014.120 24961629 PMC4205099

[5] LaFramboise T. Single nucleotide polymorphism arrays: a decade of biological, computational and technological advances. Nucleic Acids Res 2009;37:4181-93. 10.1093/nar/gkp552 19570852 PMC2715261

[6] Smart A Bolnick DA Tutton R. Health and genetic ancestry testing: time to bridge the gap. BMC Med Genomics 2017;10:3. 10.1186/s12920-016-0240-3 28069037 PMC5223458

[7] Price AL Spencer CCA Donnelly P. Progress and promise in understanding the genetic basis of common diseases. Proc Biol Sci 2015;282:20151684. 10.1098/rspb.2015.1684 26702037 PMC4707742

[8] Visscher PM Goddard ME. From R.A. fisher’s 1918 paper to GWAS a century later. Genetics 2019;211:1125-30. 10.1534/genetics.118.301594 30967441 PMC6456325

[9] Wright CF West B Tuke M. Assessing the Pathogenicity, Penetrance, and Expressivity of Putative Disease-Causing Variants in a Population Setting. Am J Hum Genet 2019;104:275-86. 10.1016/j.ajhg.2018.12.015 30665703 PMC6369448

[10] Bycroft C Freeman C Petkova D. The UK Biobank resource with deep phenotyping and genomic data. Nature 2018;562:203-9. 10.1038/s41586-018-0579-z 30305743 PMC6786975

[11] Anderson CA Pettersson FH Clarke GM Cardon LR Morris AP Zondervan KT. Data quality control in genetic case-control association studies. Nat Protoc 2010;5:1564-73. 10.1038/nprot.2010.116 21085122 PMC3025522

[12] van der Wouden CH Carere DA Maitland-van der Zee AH Ruffin MT 4th Roberts JS Green RC Impact of Personal Genomics Study Group. Consumer Perceptions of Interactions With Primary Care Providers After Direct-to-Consumer Personal Genomic Testing. Ann Intern Med 2016;164:513-22. 10.7326/M15-0995 26928821

[13] Horton R Crawford G Freeman L Fenwick A Wright CF Lucassen A. Direct-to-consumer genetic testing. BMJ 2019;367:l5688. 10.1136/bmj.l5688 31619392 PMC6829432

[14] Schleit J Naylor LV Hisama FM. First, do no harm: direct-to-consumer genetic testing. Genet Med 2019;21:510-1. 10.1038/s41436-018-0071-z 29904164

[15] Moscarello T Murray B Reuter CM Demo E. Direct-to-consumer raw genetic data and third-party interpretation services: more burden than bargain? Genet Med 2019;21:539-41. 10.1038/s41436-018-0097-2 29997392 PMC6752274

[16] Tandy-Connor S Guiltinan J Krempely K. False-positive results released by direct-to-consumer genetic tests highlight the importance of clinical confirmation testing for appropriate patient care. Genet Med 2018;20:1515-21. 10.1038/gim.2018.38 29565420 PMC6301953

[17] Esplin E Haverfield E Yang S Herrera B Anderson M Nussbaum RL. Limitations of HBOC Direct-To-Consumer Genetic Screening: False Positives, False Negatives and Everything in Between. Cancer Res 2019;79(4 Suppl):P4-03-06.

[18] MIT Technology Review. More than 26 million people have taken an at-home ancestry test. 2019. https://www.technologyreview.com/s/612880/more-than-26-million-people-have-taken-an-at-home-ancestry-test/.

[19] Ball MP Bobe JR Chou MF. Harvard Personal Genome Project: lessons from participatory public research. Genome Med 2014;6:10. 10.1186/gm527 24713084 PMC3978420

[20] Van Hout CV Tachmazidou I Backman JD Geisinger-Regeneron DiscovEHR Collaboration Regeneron Genetics Center. Exome sequencing and characterization of 49,960 individuals in the UK Biobank. Nature 2020;586:749-56. 10.1038/s41586-020-2853-0 33087929 PMC7759458

[21] Lek M Karczewski KJ Minikel EV Exome Aggregation Consortium. Analysis of protein-coding genetic variation in 60,706 humans. Nature 2016;536:285-91. 10.1038/nature19057 27535533 PMC5018207

[22] Abecasis GR Auton A Brooks LD 1000 Genomes Project Consortium. An integrated map of genetic variation from 1,092 human genomes. Nature 2012;491:56-65. 10.1038/nature11632 23128226 PMC3498066

[23] Landrum MJ Lee JM Benson M. ClinVar: public archive of interpretations of clinically relevant variants. Nucleic Acids Res 2016;44(D1):D862-8. 10.1093/nar/gkv1222 26582918 PMC4702865

[24] Thorvaldsdóttir H Robinson JT Mesirov JP. Integrative Genomics Viewer (IGV): high-performance genomics data visualization and exploration. Brief Bioinform 2013;14:178-92. 10.1093/bib/bbs017 22517427 PMC3603213

[25] Mattocks CJ Morris MA Matthijs G EuroGentest Validation Group. A standardized framework for the validation and verification of clinical molecular genetic tests. Eur J Hum Genet 2010;18:1276-88. 10.1038/ejhg.2010.101 20664632 PMC3002854

[26] Cohen JF Korevaar DA Altman DG. STARD 2015 guidelines for reporting diagnostic accuracy studies: explanation and elaboration. BMJ Open 2016;6:e012799. 10.1136/bmjopen-2016-012799 28137831 PMC5128957

[27] Wuttke TV Jurkat-Rott K Paulus W Garncarek M Lehmann-Horn F Lerche H. Peripheral nerve hyperexcitability due to dominant-negative KCNQ2 mutations. Neurology 2007;69:2045-53. 10.1212/01.wnl.0000275523.95103.36 17872363

[28] Vasen HFA Möslein G Alonso A. Guidelines for the clinical management of Lynch syndrome (hereditary non-polyposis cancer). J Med Genet 2007;44:353-62. 10.1136/jmg.2007.048991 17327285 PMC2740877

[29] Kapoor RR Flanagan SE Arya VB Shield JP Ellard S Hussain K. Clinical and molecular characterisation of 300 patients with congenital hyperinsulinism. Eur J Endocrinol 2013;168:557-64. 10.1530/EJE-12-0673 23345197 PMC3599069

[30] Chin ELH da Silva C Hegde M. Assessment of clinical analytical sensitivity and specificity of next-generation sequencing for detection of simple and complex mutations. BMC Genet 2013;14:6. 10.1186/1471-2156-14-6 23418865 PMC3599218

[31] Goldstein JI Crenshaw A Carey J Swedish Schizophrenia Consortium ARRA Autism Sequencing Consortium. zCall: a rare variant caller for array-based genotyping: genetics and population analysis. Bioinformatics 2012;28:2543-5. 10.1093/bioinformatics/bts479 22843986 PMC3463112

[32] Nelson SC Bowen DJ Fullerton SM. Third-Party Genetic Interpretation Tools: A Mixed-Methods Study of Consumer Motivation and Behavior. Am J Hum Genet 2019;105:122-31. 10.1016/j.ajhg.2019.05.014 31204012 PMC6612532

[33] van de Putte R Wijers CHW Reutter H. Exome chip association study excluded the involvement of rare coding variants with large effect sizes in the etiology of anorectal malformations. PLoS One 2019;14:e0217477. 10.1371/journal.pone.0217477 31136621 PMC6538182

[34] Chen R Shi L Hakenberg J. Analysis of 589,306 genomes identifies individuals resilient to severe Mendelian childhood diseases. Nat Biotechnol 2016;34:531-8. 10.1038/nbt.3514 27065010

[35] Border R Johnson EC Evans LM. No Support for Historical Candidate Gene or Candidate Gene-by-Interaction Hypotheses for Major Depression Across Multiple Large Samples. Am J Psychiatry 2019;176:376-87. 10.1176/appi.ajp.2018.18070881 30845820 PMC6548317

[36] Kellog G, Bisignano A, Jaremko M, Puig O. Implications of FDA Approval for Genetic Tests of Limited Clinical Utility. 2019. https://acmg.expoplanner.com/index.cfm?do=expomap.sess&event_id=13&session_id=8826.

[37] Ludwig KK Neuner J Butler A Geurts JL Kong AL. Risk reduction and survival benefit of prophylactic surgery in BRCA mutation carriers, a systematic review. Am J Surg 2016;212:660-9. 10.1016/j.amjsurg.2016.06.010 27649974

