# Supplementary material for: Use of SNP chips to detect rare pathogenic variants: retrospective, population based diagnostic evaluation
Source: BMJ. 2021 Feb 15;372:n214. doi: 10.1136/bmj.n214 (PMC7879796; doi:10.1136/bmj.n214)

Supplementary Figure A PPV of UK Biobank BiLEVE® SNP-chip for detecting variants at different population frequencies.

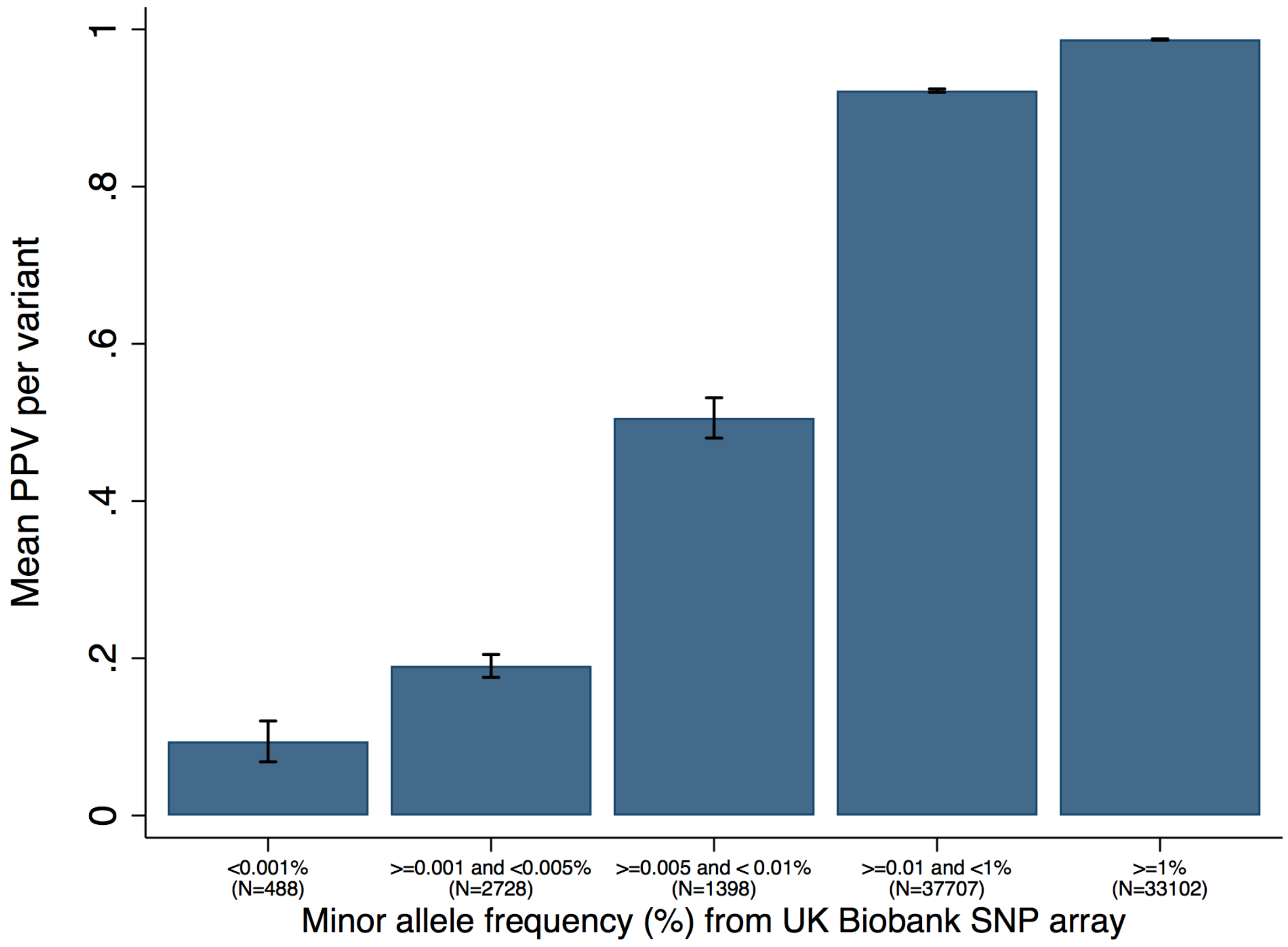

Supplementary Figure B PPV of SNP chips available through the Personal Genome Project for detecting variants at different population frequencies.

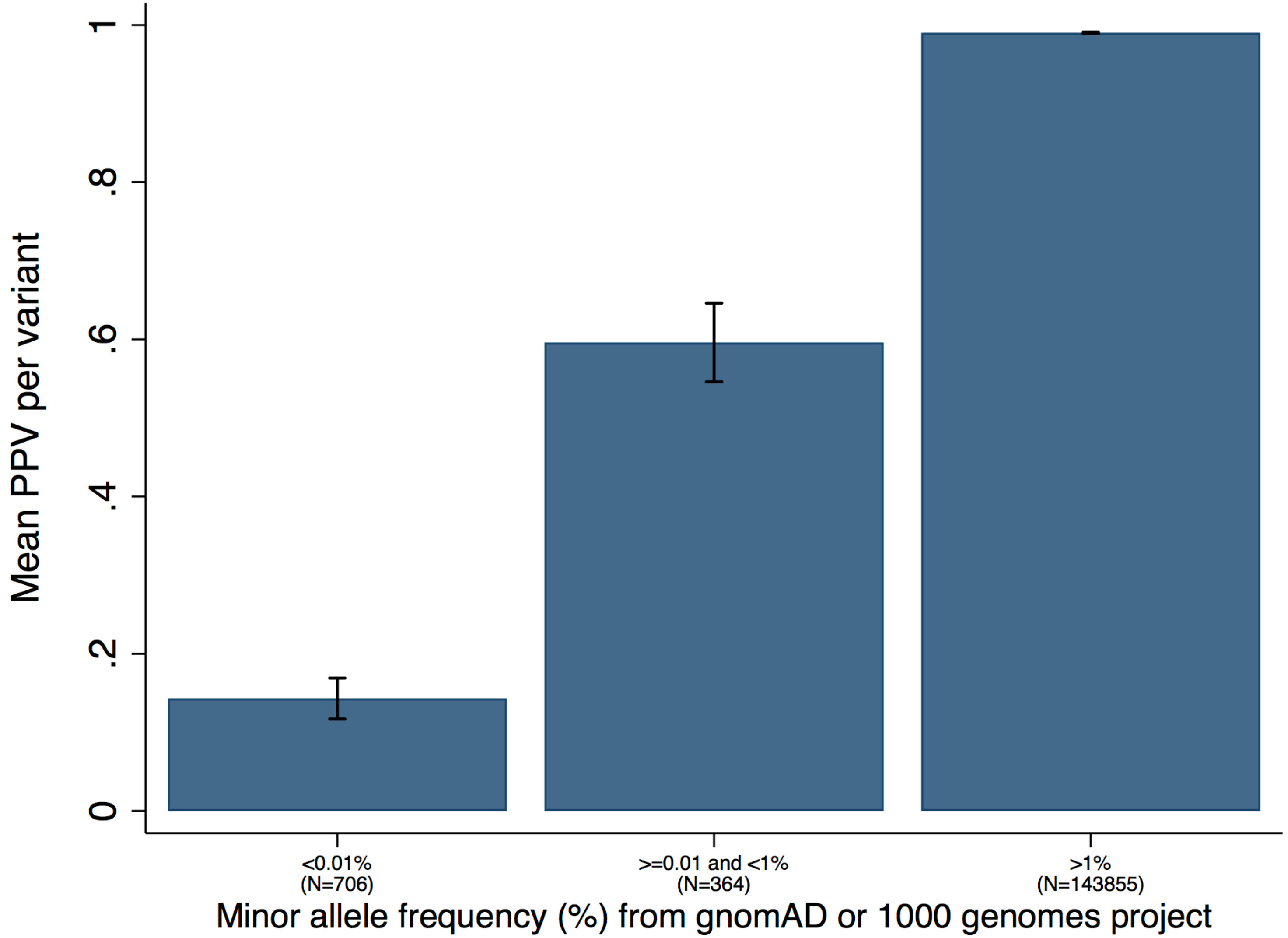

Supplementary Figure C STARD diagram to report flow of participants with a pathogenic BRCA variant on the UK Biobank BiLEVE® SNP chip compared with sequencing.

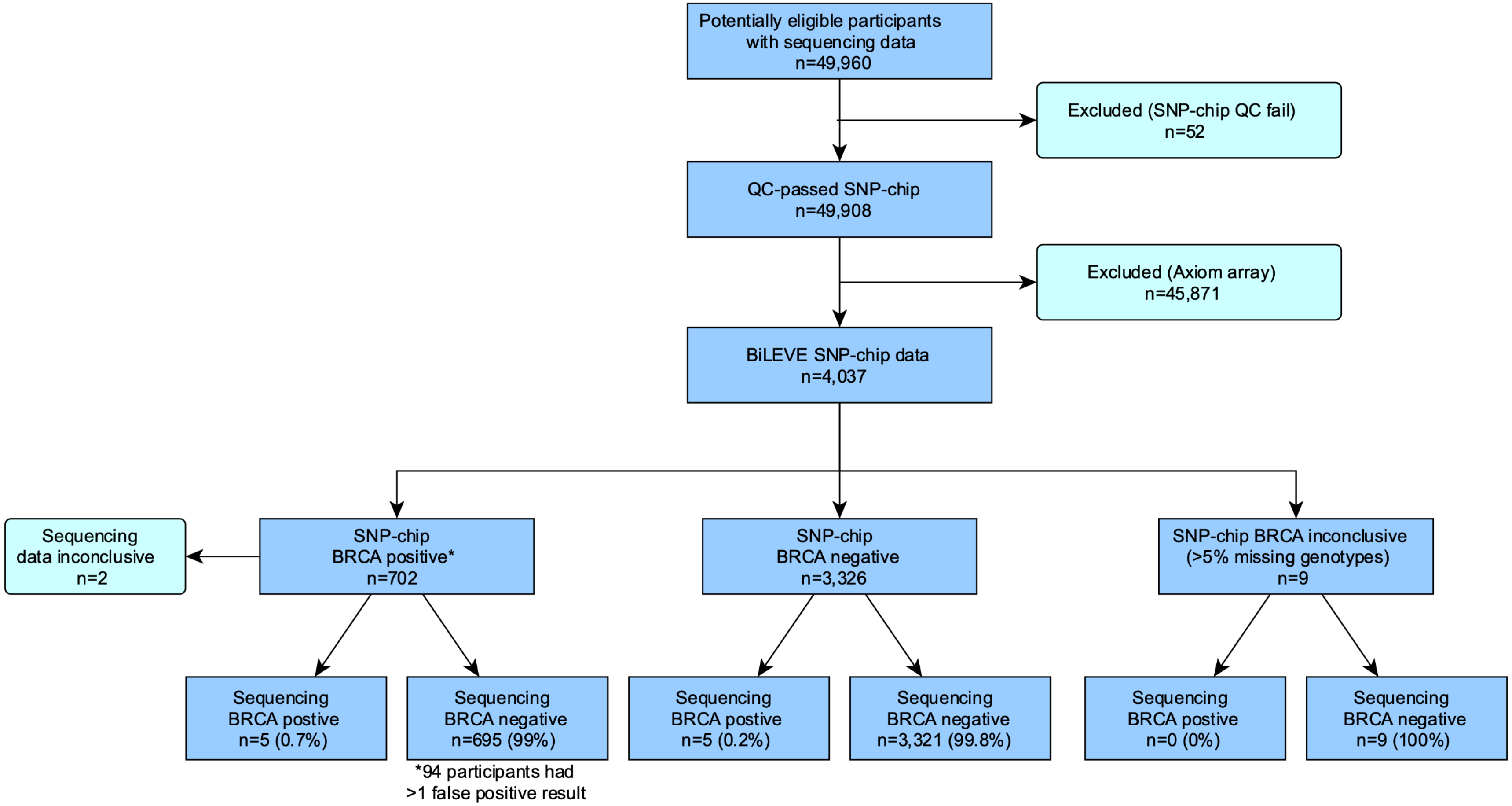

Supplement: Supplementary file 1 — Web appendix: Supplementary figures [file weem054760.ww1.pdf]
